# Drug Resistance of CPT-11 in Human DLD-1 Colorectal Cancer Cells through MutS Homolog 2 Upregulation

**DOI:** 10.7150/ijms.52620

**Published:** 2021-01-16

**Authors:** Ko-Chao Lee, Chia-Kung Yen, Cheng-Nan Chen, Shun-Fu Chang, Ying-Chen Lu, Wen-Shih Huang

**Affiliations:** 1 Department of Colorectal Surgery, Department of Surgery, Chang Gung Memorial Hospital; Kaohsiung Medical Center, Kaohsiung 833, Taiwan.; 2 Department of Food Science, National Chiayi University, Chiayi 600, Taiwan.; 3 Department of Biochemical Science and Technology, National Chiayi University, Chiayi 600, Taiwan.; 4 Department of Medical Research and Development, Chiayi Chang Gung Memorial Hospital, Chiayi 613, Taiwan.; 5 Graduate Institute of Clinical Medical Sciences, College of Medicine, Chang Gung University, Taoyuan 333, Taiwan.; 6 Division of Colon and Rectal Surgery, Department of Surgery, Chiayi Chang Gung Memorial Hospital, Chiayi 613, Taiwan.

**Keywords:** Colorectal cancer, CPT-11, DNA topoisomerases, Drug resistance, MutS homolog 2

## Abstract

Colorectal cancers (CRCs) is the most commonly diagnosed and deadly cancer types in the world. Despite advances in chemotherapy for CRCs, drug resistance remains a major challenge to high incurable and eventually deadly rates for patients. CPT-11 is one of the current chemotherapy agents for CRC patients and the CPT-11 resistance development of CRCs is also inevitable. Recently, accumulating data has suggested that DNA repair system might be an inducer of chemotherapy resistance in cancer cells. Thus, this study was aimed to examine whether MutS homolog (MSH) 2, one member of DNA repair system, plays a role to affect the cytotoxicity of CPT-11 to CRCs. Human DLD-1 CRC cells were used in this study. It was shown that MSH2 gene and protein expression could be upregulated in DLD-1 cells under CPT-11 treatment and this upregulation subsequently attenuates the sensitivity of DLD-1 cells to CPT-11. Moreover, ERK1/2 and Akt signaling and AP-1 transcription factor have been found to modulate these effects. These results elucidate the drug resistance role of MSH2 upregulation in the CPT-11-treated DLD-1 CRC cells. Our findings may provide a useful thought for new adjuvant drug development by controlling the DNA repair system.

## Introduction

Colorectal cancer (CRC) is one of the most commonly diagnosed and deadly cancer types for both men and women in the world 1. In spite of extensive recommendations for preventive examination in many countries, including Taiwan, the high percentage of CRC patients are still presented with advanced and metastatic stages and their survival rates are still low 2. Moreover, despite advances in chemotherapy for CRC, the development of drug resistance remains one of challenges to high incurable and eventually deadly rates for patients. Hence, further investigation of early diagnostic markers and resistance mechanism of chemotherapy are still urgently needed to the improvement of clinical theranostics and practice for CRC patients.

DNA topoisomerases, including type I, II, and III, are the nuclear enzymes that could open the supercoiled structure of DNA by creating a transient cleavage of DNA single (topoisomerases I and III) or double (topoisomerases II) strand and be therefore involved in initiating the DNA replication, repair, recombination, and transcription 3-5. Thus, it has been proposed that blocking the topoisomerase activity may be one of the therapeutic targets to anticancer 4-7. CPT-11, a camptothecin derivate, has been demonstrated to have a potential anticancer activity for many solid cancers, including the CRC 8-10. Its cytotoxic mechanism of anticancer is initiated by blocking the DNA topoisomerase I activity, which binds and stabilizes the covalent structure of topoisomerase I-DNA complex and subsequently convert the DNA single strand break to double strand break. The formation of DNA double strand break could subsequently result in the G2/M cell cycle arrest and consequent cell death 8-10. CPT-11 is a kind of prodrug; in the cells, CPT-11 will be converted to an active metabolite, i.e., SN-38. It has been verified that the anticancer activity of SN-38 is over 100- to 1000-fold than its parent drug 11-12. Currently, CPT-11 has already been extensively used in clinical CRC single and/or combined therapy. However, the resistance of CPT-11 in CRC has also been reported. The resistance mechanisms include (*i*) the *in vivo* production of active SN-38 is low, (*ii*) gene mutation and/or decreased expression of topoisomerase I, and (*iii*) changes in SN-38 binding activity to topoisomerase I-DNA complex 2. In this study, the possible role of DNA repair enzyme in CPT-11 resistance development of CRC has been examined.

Mismatch repair (MMR) is one type of DNA repair enzyme systems; it could correct the incorrect base-pairing, unmatched DNA loop, and DNA single/double stand break generated during the DNA replication/recombination and hence stabilize the genome structure. The enzymes in MMR system, e.g., MutL homolog (MLH) 1/3 and MutS homolog (MSH) 2/3/6, function by heterodimerization 13-14. CRC development has been proposed to have two types of pathways, i.e., chromosomal instability (CIN, ~85% incidence) and deficient mismatch repair (dMMR, ~15% incidence) pathways 15-16. The development of dMMR CRC is because of the mutation and inactivation of MMR enzymes in cancer cells. However, recent studies have also suggested that unregulated expression of MMR enzymes in cancer cells might also contribute to the resistance of chemotherapeutic drugs 15-20. Moreover, it has also been reported that differential MMR enzymes mutations in different CRC cell lines could affect their sensitivity to DNA topoisomerases inhibitors, including CPT-11 21. Thus, according to these findings, it has been indicated that the precise expression and activation of MMS enzymes in cells play critical role in balancing normal cell physiology, carcinogenesis, and drug resistance to chemotherapeutic agents. Further elucidating the role and mechanism of MMR enzymes in CRC is still important in benefiting future clinical treatment.

In the present study, we aimed to determine the possible role of MMR enzymes in cytotoxicity of CPT-11 to CRC cells. It was shown that CPT-11 activates transcription factor AP-1 to regulate MSH2 expression through ERK1/2 and Akt signaling in CRC DLD-1 cells and this MSH2 induction could further attenuate the sensitivity of DLD-1 to CPT-11. Our findings provide new insights into the role and mechanism of MSH2 upregulation in the resistance development of CRC cells to CPT-11.

## Material and Methods

### Materials

The materials for cell culture were purchased from Gibco (Grand Island, NY, USA). MAPK inhibitor, including PD98059 for ERK1/2, SP600125 for JNK, SB203580 for p38, and LY294002 for Akt, and AP-1 inhibitor, i.e., Tanshinone IIA, were purchased from Sigma (St. Louis, MO). Rabbit antibodies against MSH2, phosphor-ERK1/2, ERK1/2, phosphor-Akt, Akt, and β-actin were purchased from Cell Signaling Technology (Beverly, MA). The control-, MSH2-, and c-jun-siRNA were purchased from Thermo (Waltham, MA). All others chemicals were obtained from Sigma (St. Louis, MO).

### Cell culture

The DLD-1 CRC cells were purchased from the cell bank of the Taiwan Food Industry Research and Development Institute (Hsinchu, Taiwan). DLD-1 cells were cultured in Dulbecco's Modified Eagle Medium supplemented with 10% FBS and 1% penicillin/streptomycin in a 5% CO_2_ incubator (37°C).

### MTT assay

DLD-1 cells were seeded in 96-well plates. The cell survival/death was ezamined by the MTT (3-(4,5-dimethylthiazol-2-yl)-2,5-diphenyltetrazolium bromide) assay. After treatment, MTT solution (0.5 mg/mL) was added to the wells and further incubated for 3 h at 37ºC to allow MTT reduction. Adding the DMSO to dissolve the formazan crystals and the absorbance was measured at 570 nm with a spectrophotometer.

### Real-time quantitative PCR

The RNA was extracted by Trizol kit and was converted to cDNA by Reverse-transcription kit. Real-time PCR assay of the indicated genes was performed by using the FastStart DNA SYBR Green I kit (Thermo, Waltham, MA). The primers of the indicated genes were: MSH2 (positive: 5^′^-AAGCCCAGGATGCCATTG-3^′^; negative: 5′-CATTTGACACGTGAGCAAAGC-3′) and GAPDH (positive: 5′-AGGTGAAGGTCGGAGTCAAC-3′; negative: 5′-CCATGTAGTTGAGGTCAATGAAGG-3′). GAPDH gene was the internal control. The RT-PCR were determined in duplicate.

### Western blot analysis

DLD-1 cells were lysed with lysis buffer (1% NP-40, 0.5% sodium deoxycholate, 0.1% SDS, and a protease/phosphatase inhibitor cocktail). Protein concentration was examined by using the Bio-Rad protein assay kit (Bio-Rad, Hercules, CA). Equal amounts of protein samples (45 µg) were separated by SDS-polyacrylamide gel electrophoresis (PAGE) (10% running, 4% stacking), transferred to nitrocellular paper, and detected by adding the designated primary and horseradish peroxidase-conjugated secondary antibodies.

### siRNA transfection

DLD-1 cells were cultured in DMEM supplemented with 10% FBS (no antibiotics) in a 5% CO_2_ incubator (37°C) overnight and then transfected with the control-, MSH2-, or c-jun-specific siRNA by using an RNAiMAX transfection reagent (Thermo, Waltham, MA). After 48 h incubation, the transfected cells were used in the experiments.

### AP-1 binding activity ELISA assays

The nuclear proteins were extracted by the nuclear protein extract kit (Panomics, Redwood City, CA), and the promoter binding activity of AP-1 was examined by TF ELISA kits (Panomics, Redwood City, CA). Briefly, an oligonucleotide containing an AP-1 binding site was immobilized on 96-well plate. The extracted nuclear proteins were added to the wells and the AP-1/DNA complex was detected by adding the AP-1-specific antibody and then analyzed by using a spectrophotometry.

### Luciferase assay

AP-1 transcription activity was determined by the luciferase assay. Adenoviral-AP-1-Luc vector was purchased form the Vector Biolabs, Philadelphia, PA. After infection, the AP-1 luciferase activity was analyzed by indicated method and normalized by the total protein concentrations of DLD-1 cells 29.

### Statistical analysis

The results were shown with mean ± standard error of the mean. Statistical analysis was measured by an independent Student t-test for two groups of data and analysis of variance (ANOVA) followed by Scheffe's test for multiple comparisons. *P* values < 0.05 were indicated as significant.

## Results

### CPT-11 induced MSH2 mRNA and protein expressions in a dose- and time-dependent manner in DLD-1 cells

**Figure 1:**
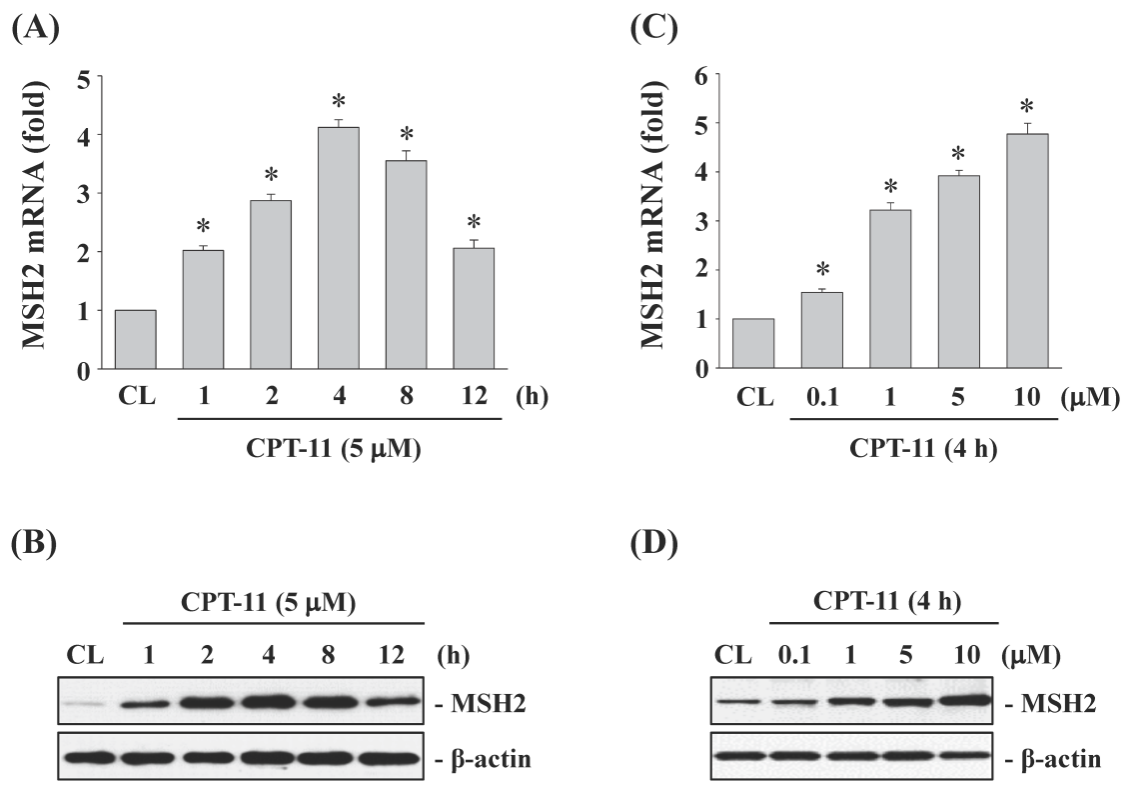
** CPT-11 induced MSH2 mRNA and protein expressions in a dose- and time-dependent manner in DLD-1 cells.** DLD-1 cells were kept as control (CL) or treated with CPT-11 (0.1, 1, 5, and 10 μM) for the indicated times. The mRNA (*A and C*) and protein (*B and D*) expression of MSH2 were determined by real-time PCR and Western blotting, respectively. Data in (*A and C*) are shown as mean ± SEM from three independent experiments. *P < 0.05 versus CL. Results in (*B and D*) are representative of three independent experiments with similar results.

To study the effects of CPT-11 on MSH2 expression in CRC cells, DLD-1 cells were kept as control or treated with CPT-11 with 5 μM for 1, 2, 4, 8, and 12 h, or with different doses (0, 0.1, 1, 5, and 10 μM) for 4 h. The changes in mRNA and protein expression of MSH2 were determined by real-time PCR and Western blotting, respectively. The MSH2 mRNA and protein levels in DLD-1 cells began to increase within 1 h of CPT-11 treatment compared to untreated control, which reached to a maximal level at 4 h and then declined but remained elevated than untreated control after 8 and 12 h of treatment (Fig. 1A and 1B). The CPT-11 dose experiments also showed that MSH2 mRNA and protein expressions could be upregulated in a dose-dependent manner in DLD-1 cells compared to untreated control (Fig. 1C and 1D).

### Gene knockdown of MSH2 in DLD-1 cells enhances the cytotoxicity induced by CPT-11

**Figure 2:**
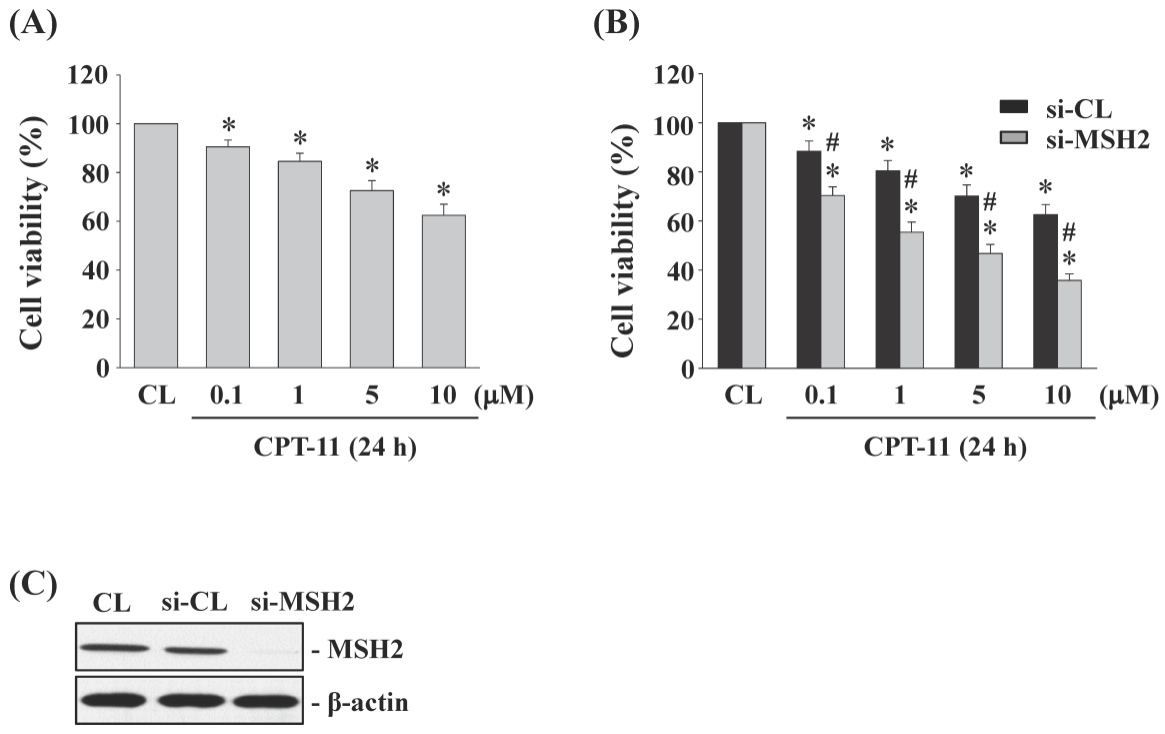
** Gene knockdown of MSH2 in DLD-1 cells enhances the cytotoxicity induced by CPT-11.** (*A*) DLD-1 cells were kept as control (CL) or treated with different doses of CPT-11 for 24 h and the cell viability was assayed by the MTT test. (*B-C*) DLD-1 cells were transfected with control (si-CL)- or MSH2 (si-MSH2)-specific siRNA and then kept as control (CL) or treated with different doses of CPT-11 for 24 or 48 h. (*B*) Cell viability was assayed by the MTT test. (*C*) The knockdown efficiency of MSH2 gene was determined by Western blotting. Data in (*A-B*) are shown as mean ± SEM from three independent experiments. *P < 0.05 versus CL. ^#^*P* < 0.05 versus si-CL/CPT-11-treated cells. Results in (*C*) are representative of three independent experiments with similar results.

DLD-1 cells were kept as control or treated with different doses of CPT-11 (0, 0.1, 1, 5, and 10 μM) for 24 h and then the cell survival was analyzed by MTT assay. Cells stimulated with CPT-11 resulted in a significant decrease in the cell survival of DLD-1 in a dose-dependent manner compared to untreated control (Figure 2A). To further determine the upregulated role of MSH2 in CPT-11-stimulated DLD-1 cells, MSH2 genes in DLD-1 cells were knocked down by using MSH2-specific siRNA. It was shown that MSH2 gene knockdown in CPT-11-stimulated cells enhances the cytotoxicity of CPT-11 in DLD-1 cells compared to the control-siRNA-transfected cells) (Figure 2B). The efficiency of the gene knockdown was confirmed because MSH2-specific siRNA caused an over 80% reduction in MSH2 protein expression in DLD-1 cells compared with untreated control and control siRNA (Figure 2C, 25 nM).

### ERK1/2 and Akt signaling regulate CPT-11-increased MSH2 expression and subsequent cell cytotoxicity of DLD-1 cells

**Figure 3:**
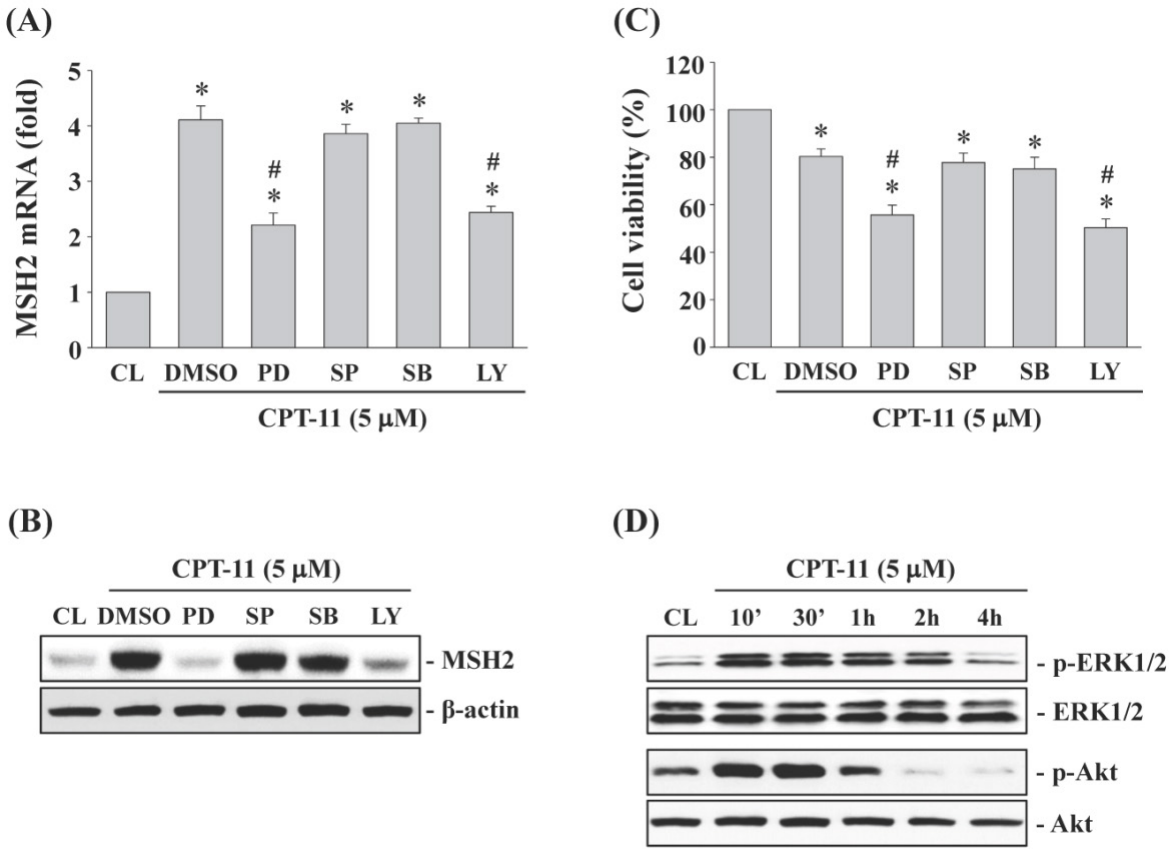
** ERK1/2 and Akt signaling regulate CPT-11-increased MSH2 expression and subsequent cell cytotoxicity of DLD-1 cells.** (*A-C*) DLD-1 cells were kept as control (CL) or pretreated with DMSO or specific kinase inhibitors for ERK1/2 (PD98059, 30 μM), JNK (SP600125, 20 μM), p38 (SB203580, 10 μM) or Akt (LY294002, 20 μM) for 1 h and then treated with CPT-11 (5 μM) for 4 and 24 h. The mRNA (*A*) and protein (*B*) expression of MSH2 were determined by real-time PCR and Western blotting, respectively. (*C*) Cell viability was assayed using the MTT test. (*D*) DLD-1 cells were kept as control (CL) or treated with CPT-11 (5 μM) for the indicated times. The ERK1/2 and Akt phosphorylations were determined by Western blotting. Data in (*A and C*) are shown as mean ± SEM from three independent experiments. *P < 0.05 versus CL. ^#^*P* < 0.05 versus DMSO/CPT-11-treated cells. Results in (*B and D*) are representative of three independent experiments with similar results.

DLD-1 cells were kept as control or pretreated with DMSO or specific kinase inhibitors for ERK1/2 (PD98059, 30 μM), JNK (SP600125, 20 μM), p38 (SB203580, 10 μM) or Akt (LY294002, 20 μM) for 1 h and then treated with CPT-11 (5 μM) for 4 and 24 h. It was shown that the activity inhibitions of ERK1/2 and Akt kinases in DLD-1 cells attenuate CPT-11-increased MSH2 mRNA (Figure 3A) and protein (Figure 3B) expressions and further decrease the cell survival of DLD-1 cells (Figure 3C). The activity inhibitions of JNK and p38 kinases did not affect the MSH2 expression and cell death of CPT-11 induction in DLD-1 cells. The phosphorylations of ERK1/2 and Akt kinases in DLD-1 cells were induced in a time-dependent manner after 10 min of CPT-11 stimulation compared to untreated control (Figure 3D).

### CPT-11 activates AP-1 transcription activity in DLD-1 cells

**Figure 4:**
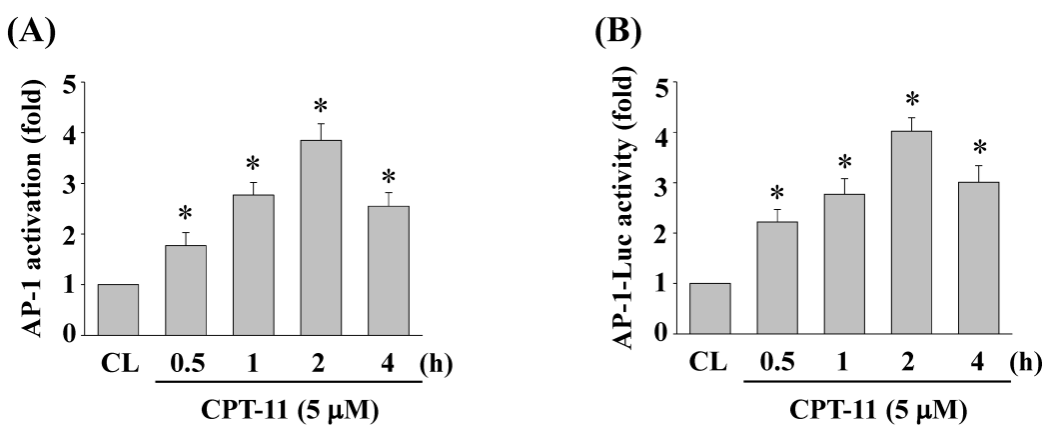
** CPT-11 activates AP-1 transcription activity in DLD-1 cells.** (*A-B*) DLD-1 cells were kept as control or treated with CPT-11 (5 μM) for the indicated times and the DNA binding activity (*A*) and transcription activity (*B*) of AP-1 were analyzed by using TF ELISA kit and luciferase assay, respectively. Data in (*A-B*) are shown as mean ± SEM from three independent experiments. *P < 0.05 versus CL.

To determine whether AP-1 could be activated in DLD-1 cells under CPT-11 stimulation, cells were kept as control or treated with CPT-11 (5 μM) for 0.5, 1, 2, and 4 h and the AP-1 DNA binding activity were analyzed by using TF ELISA kit. As shown in Figs. 4A, the DNA binding activity of AP-1 was increased in a time-dependent manner compared to untreated control. To further confirm this result, DLD-1 cells were transfected with adenovirus AP-1 luciferase reporter and then were kept as control or treated with CPT-11 (5 μM) for 0.5, 1, 2, and 4 h. The AP-1 transcription activity was analyzed by luciferase assay. As expected, it was shown that CPT-11 also significantly increases AP-1 transcription activity in a time-dependent manner (Figure 4B).

### ERK1/2 and Akt signaling regulate CPT-11-activated AP-1 transcription activity in DLD-1 cells

**Figure 5:**
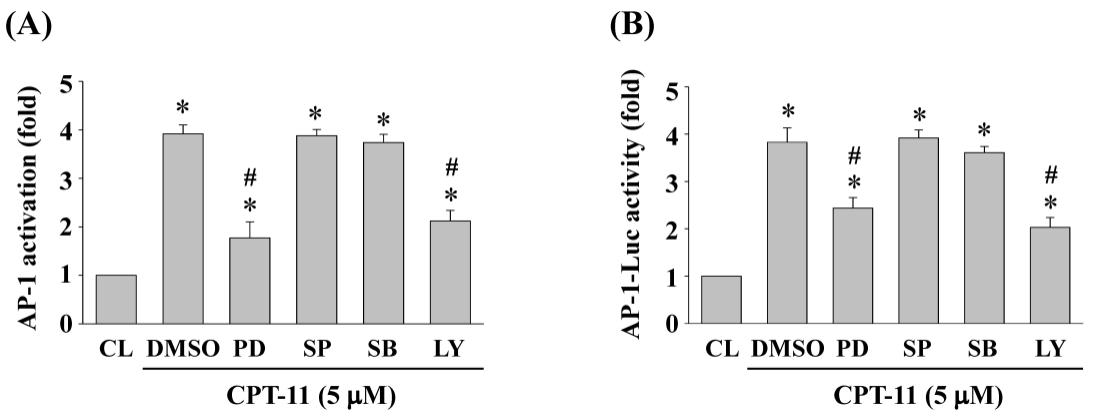
** ERK1/2 and Akt signaling regulate CPT-11-activated AP-1 transcription activity in DLD-1 cells.** (*A-B*) DLD-1 cells were kept as control or pretreated with DMSO or specific kinase inhibitors for ERK1/2 (PD98059, 30 μM), JNK (SP600125, 20 μM), p38 (SB203580, 10 μM) or Akt (LY294002, 20 μM) for 1 h and then treated with CPT-11 (5 μM) for 2 h. The DNA binding activity (*A*) and transcription activity (*B*) of AP-1 were analyzed by using TF ELISA kit and luciferase assay, respectively. Data in (*A-B*) are shown as mean ± SEM from three independent experiments. *P < 0.05 versus CL. ^#^*P* < 0.05 versus DMSO/CPT-11-treated cells.

DLD-1 cells were kept as control or pretreated with DMSO or specific kinase inhibitors for ERK1/2 (PD98059, 30 μM), JNK (SP600125, 20 μM), p38 (SB203580, 10 μM) or Akt (LY294002, 20 μM) for 1 h and then treated with CPT-11 (5 μM) for 2 h. The DNA binding activity and transcription activity of AP-1 were analyzed by using TF ELISA kit and luciferase assay, respectively. It was shown that the activity inhibitions of ERK1/2 and Akt kinases in DLD-1 cells attenuate CPT-11-increased AP-1 DNA binding activity (Figure 5A) and transcription activity (Figure 5B).

### AP-1 regulates MSH2 expression and cell survival of DLD-1 cells

**Figure 6:**
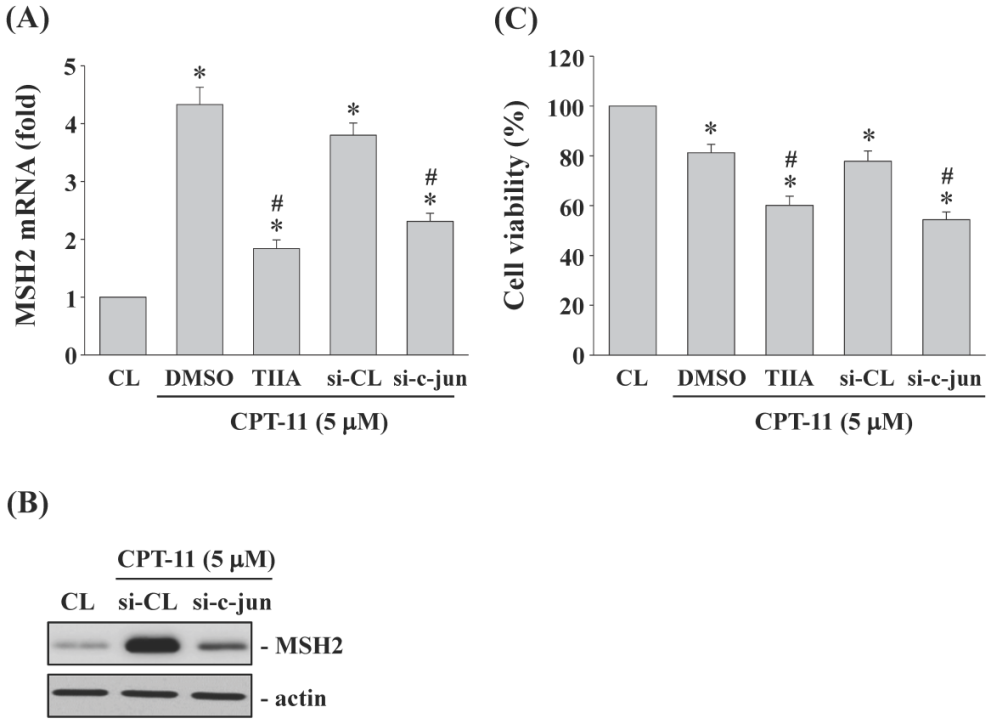
** AP-1 regulates MSH2 expression and cell survival of DLD-1 cells.** (*A-C*) DLD-1 cells were kept as control or pretreated with (*i*) DMSO or AP-1 inhibitor (Tanshinone IIA, TIIA, 3 μM) or (*ii*) control- or c-jun (si-c-jun)-specific siRNA and then treated with CPT-11 (5 μM). The mRNA (*A*) and protein (*B*) expression of MSH2 were determined by real-time PCR and Western blotting, respectively. (*C*) the cell viability was assayed using the MTT test. Data in (*A and C*) are shown as mean ± SEM from three independent experiments. *P < 0.05 versus CL. ^#^*P* < 0.05 versus DMSO or si-CL/CPT-11 treated cells. Results in (*B*) are representative of three independent experiments with similar results.

DLD-1 cells were kept as control or pretreated with (*i*) DMSO or specific inhibitor for AP-1 (Tanshinone IIA, TIIA, 3 μM) or (*ii*) control- or c-jun (subunit of AP-1)-specific siRNA and then treated with CPT-11 (5 μM) for 4 and 24 h. The MSH2 mRNA/protein expression and cell survival of DLD-1 cells were analyzed. It was shown that both AP-1 transcription activity inhibition and c-jun gene knockdown significantly decrease CPT-11-increased MSH2 mRNA (Figure 6A) and protein (Figure 6B) expressions and cell survival (Figure 6C) of DLD-1 cells.

## Discussion

**Figure 7:**
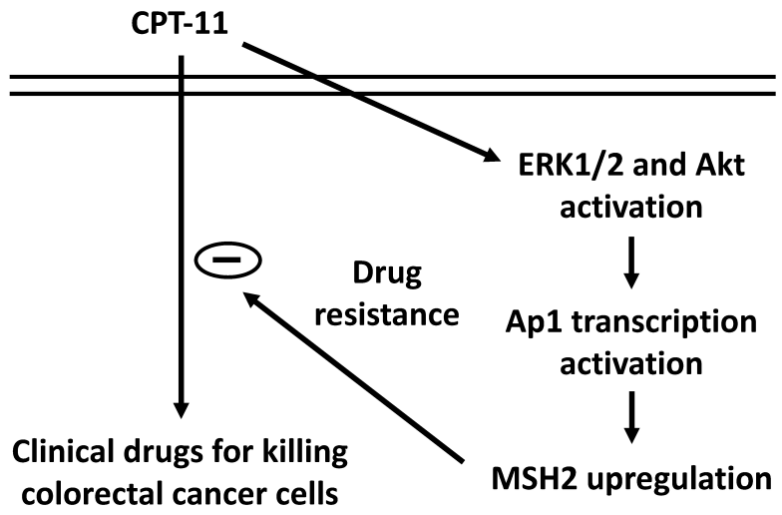
Schematic representation of the signaling pathways affecting the MSH2 upregulation of CPT-11 induction and subsequent CPT-11 resistance development in DLD-1 CRC cells.

Our systematic experiments demonstrated that (*i*) MSH2 mRNA and protein expressions could be upregulated in DLD-1 cell while CPT-11 treatment and this upregulation could hence reduce the cytotoxicity of CPT-11 in DLD-1 cells. (*ii*) ERK1/2 and Akt signaling were involved in the MSH2 upregulation and subsequent cell death of CPT-11 induction. (*iii*) AP-1 is a major transcription factor to control MSH2 expression in DLD-1 cells under CPT-11 treatment. Thus, this study has elucidated a drug resistance mechanism by which MSH2 expression could be upregulated to attenuate the sensitivity of CRC cells to CPT-11 (summarized in Figure 7).

CPT-11 is an existing chemotherapy drug most widely used in clinical medication to treat CRCs. However, the resistance development of CRC to CPT-11 is still inevitable. It has been suggested that the CPT-11 resistance might be elicited by the low levels of SN-38 and DNA topoisomerase and instability of SN-38-DNA topoisomerase structure 2. Intracellular level of SN38 could be changed by the efflux, hydrolysis, and metabolism of CPT-11, which are regulated by ATP-binding cassette transporters, carboxylesterase, and hepatic cytochrome P-450 enzymes, respectively 2, 22-26. On the other hand, the expression level and mutation of topoisomerase I gene, i.e., *TOP1*, are the major causes to affect the binding stability of SN-38 to topoisomerase I/DNA complex 27. Therefore, although the data inconsistent has still existed in the literatures, the accumulating data has indicated that the resistance of CRC to CPT-11 might be developed by the activity modulation of these enzymes and the copy number change/mutation of *TOP1*. Moreover, it should also be noted that most of these results have come from the *in vitro* studies, therefore, the further *in vivo* evidences about the CPT-11 resistance of CRCs are still needed to exam for the improvement of CPT-11 therapy of CRC patients.

Accumulating evidence has indicated that unregulated expression of DNA repair enzymes, including MMR pathway, is a potential initiator triggering the resistance development of cancer to chemotherapy and radiotherapy 19-20. Therefore, in the present study, we showed another possible mechanism of CPT-11 resistance of CRCs. Our results found that CPT-11 could upregulate MSH2 (one member of MMR pathway) gene/protein expression to reduce the cytotoxicity of CPT-11 to DLD-1 CRC cells. Moreover, this event was regulated by ERK1/2 and Akt signaling and AP-1 transcription factor. In the mammalian cells, DNA repair system plays an important role in the DNA and genome stabilization through repairing the unusual lesions, including DNA breaks or mismatch pairs, and therefore the deficient mutation of DNA repair enzymes is thought to be one of major causes for tumorigenesis 20. However, recently, it has been demonstrated that the overexpression of DNA repair enzymes in cancer cells is also harmful because unnormal increased DNA repair activities result in the attenuation of chemotherapy efficiency 19-20. CRCs is one of the typical models. It has been shown that ~15% CRC incidence is because of the deficient mismatch repair 15-16. However, one of chemotherapy resistance mechanism for CRC is induced by upregulated expression of X-ray repair cross-complementing protein 1 (XRCC1), one member of base excision repair (BER) pathway, in HCT-116 cells under 5-FU treatment 28. Thus, these results, including ours, have elucidated that appropriate modulation of DNA repair enzymes expression in cancer cells during chemotherapy proceeding might be a potential strategy to enhance the chemotherapy efficiency through reducing the development of drug resistance.

Previous study has found that different types of CRC cells which have different endogenous levels of MMR enzymes have different drug sensitivity to DNA topoisomerase inhibitors, including CPT-11 and etoposide 21. Our results further demonstrated that CPT-11 treatment could also affect the unnormal overexpression of MSH2 in CRC cells and elucidated the underlying mechanism. However, the limitations are (*i*) we have not completed the animal and clinical test in the present study, which is still an ongoing project in our lab; and (*ii*) in the clinical medication, CRC patients might be treated with the CPT-11/5-FU combined therapy. However, as the previous description, 5-FU resistance of CRCs might be induced by upregulating the XRCC1 expression. Hence, the drug resistance mechanism through upregulating DNA repair enzymes expression under patients treated with combined therapy also needs to be further discussed.

The presented study demonstrates that CPT-11, an important and current clinical drug, could activate the ERK1/2 and Akt signaling and AP-1transcription factor to upregulate the MSH2 expression and hence consequently decrease the cytotoxicity of CPT-11 to CRC cells. These findings provide new insight into the understanding of possible drug resistance mechanism of CRC treated with CPT-11 and provide a useful thought for new adjuvant drug development through controlling the DNA repair system.

## Abbreviations

CRC: colorectal cancer
MMR: mismatch repair
MLH: MutL homolog
MSH: MutS homolog
CIN: chromosomal instability
dMMR: deficient mismatch repair
XRCC1: X-ray repair cross-complementing protein 1
BER: base excision repair
MTT: 3-(4,5-dimethylthiazol-2-yl)-2,5-diphenyltetrazolium bromide
PAGE: SDS-polyacrylamide gel electrophoresis
